# Neither MRI, CT nor US is superior to diagnose tumors in the salivary glands – an extended case study

**DOI:** 10.1186/1746-160X-3-19

**Published:** 2007-04-03

**Authors:** Claudia Rudack, Sabine Jörg, Stephan Kloska, Wolfgang Stoll, Oliver Thiede

**Affiliations:** 1 Department of Otorhinolaryngology, Head and Neck Surgery, University Hospital Münster, Germany; 2 Department of Clinical Radiology, University Hospital Münster, Germany

## Abstract

**Objectives:**

Ultrasonography (US), computed tomography (CT) and magnetic resonance imaging (MRI) are the most common radiological procedures for the diagnosis of tumor-like lesions of the salivary glands. The aim of the present study was to determine whether MRI or CT provide additional information besides that delivered by US.

**Study design/Methods:**

109 patients with a tumor-like lesion of the salivary glands underwent surgery. MRI and CT were arranged in 73 and in 40 patients respectively, whereas all 109 patients were prospectively diagnosed by US. The results of CT, MRI and US were compared with the histological outcome. Furthermore, the recent rise in the number of CT and MRI studies was investigated.

**Results:**

On CT and MRI, there was no rise in the percentage of malignant tumors or advanced surgical procedures. In respect of the radiological assessment of the lesion (benign/malignant) and the correct diagnosis, CT, MRI and US were comparable in terms of sensitivity, specificity and accuracy. No significant difference was found in the Chi-square test (p > 0.05).

**Conclusion:**

The evaluation of the preoperative results of CT, MRI and US revealed no advantage for CT or MRI; these procedures are only required in specific cases. An update or revision of the current preoperative diagnostic management is deemed necessary.

## Background

**Figure 1:**
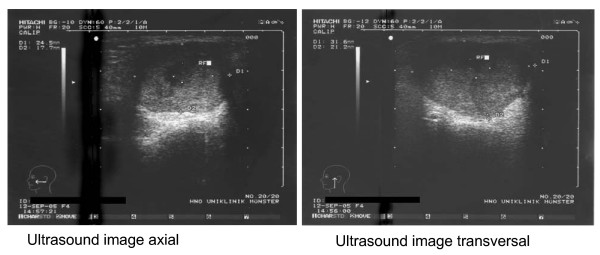
Pleomorphic Adenoma of the left parotid gland: Ultrasound image axial and Ultrasound image transversal

Tumor-like lesions of the salivary glands constitute 3% to 6% of all head-and-neck tumors. Besides clinical examination (palpation), salivary gland tumors – malignant or benign – are diagnosed by imaging procedures such as computed tomography (CT), magnetic resonance imaging (MRI) or ultrasonography (US); sialography has become less popular. Most guidelines of ENT task forces [2] recommend ultrasound as the initial imaging modality of choice for the assessment of palpable abnormalities of the salivary gland. US is able to demonstrate benign and malignant features of focal lesions and can be used to guide fine-needle aspiration biopsy or core biopsy to confirm their benign or malignant nature (Figure 1). Furthermore, US can be used to establish the need for imaging procedures (CT or MRI), particularly in those lesions showing malignant features on ultrasonography, or large masses whose extent is difficult to assess with US, particularly if located in the deep lobe [8,14][16] .

CT is also useful for the detection of tumors and the assessment of tumor extent. However, CT is limited with regard to the prediction of histopathological characteristics. Although irregular tumor margins or invasion into adjacent structures on CT suggests malignancy, a benign tumor may well mimic a malignant lesion on CT.

**Figure 2:**
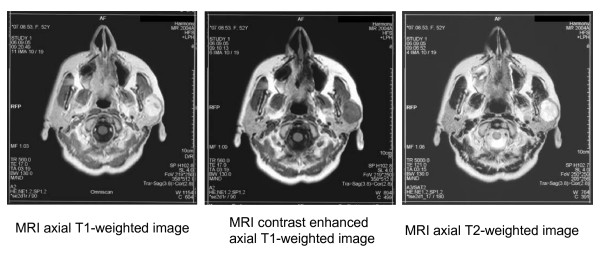
Pleomorphic Adenoma of the left parotid gland: MRI axial T1-weighted image, MRI contrast enhanced axial T1-weighted image and MRI axial T2-weighted image

In the last decade, technical advancements in the CT technique have extended the value of this procedure for the detection and characterization of tumors in regions others than salivary glands. So far, neither MRI nor CT was found to be superior in the prediction of the histomorphology of tumors in the salivary glands (Figure 2). Some studies found that MRI clearly outweighs CT in this regard [3]. A study published by Konyuncu et al. in 2003 revealed that CT and MRI provide nearly the same information for pre-surgical planning and diagnosis [12]. Freling et al. pointed out that malignant tumors are marked by erosion of surrounding bone, which is better visualized on CT [5][6].

The purpose of this study was to investigate the capability of different imaging procedures such as US, MRI and CT of the recent generation to predict the nature (benign/malignant) and the presumptive diagnosis of palpable tumors in the salivary glands. The results were compared with those of histomorphological studies performed after the surgical procedure. Furthermore, the use of imaging modalities as a diagnostic tool in salivary gland tumors by ENT-specialists and general practitioners were investigated.

## Materials and methods

### Study population

582 patients with palpable tumor of the salivary glands were referred to our outpatient department between January 2000 and November 2004 for further diagnosing and treatment. The total number of scans – MRI or CT – performed in 582 patients during the years 2000–2004, prior to the visit in the outpatient care, has been assessed. In order to compare the quality of the different scans the investigations had to fulfil the guidelines and technical standards of the American College of Roentgenology (ACR) [1] (see below).

All patients with standard scans were investigated additionally, prospectively by an ultrasound in our outpatient department. 109 of these patients who already had undergone a CT, a MRI, or both according to the guidelines and technical standards of the American College of Roentgenology (ACR), before their first examination at the ENT-department were enrolled in this study (in accordance with the current version of the 1964 Declaration of Helsinki). In 20 patients of these 109 patients (48 female, 44%; 61 male, 56%; mean age 54.9 years) the submandibular gland was affected while in 89 cases the tumor was located in the parotid gland.

In 67/582 patients, CTs and MRIs did not meet the criteria of ACR. These patients were not included to the study.

### CT and MRI inclusion criteria

The CT and MRI investigations were performed by radiologists in private practices or by radiology departments of different hospitals. The investigations had to fulfil the guidelines and technical standards of the American College of Roentgenology (ACR) [1].

The criteria were as follows:

1. The reports of the CT and MRI examination had to provide information about the technical equipment and the parameters used to perform the scan.

2. For CT examination, a transverse scan with intravenous contrast and a slice thickness of 5 mm or less were mandatory. A soft tissue algorithm was required for the reconstruction.

3. For the MRI examination, the protocol consisted of a T1-weighted sequence before and after intravenous contrast, as well as a T2-weighted sequence. Although it was not mandatory, a fat-suppressed T1-weighted sequence after contrast application was preferred; this was performed in 52 cases (70.3%).

4. The reports of the CT or the MRI examination had to include a statement about the nature (benign/malignant) and the presumptive diagnosis of tumor histomorphology (e.g., cystadenolymphoma).

5. The field strength of MRI had to be 1.0 or 1.5 Tesla, and a standard head coil had to be employed to minimize the influence of technical factors.

The files were scrutinized to determine whether a general practitioner or an otolaryngologist had ordered the CT/MRI examination.

### Ultrasound

After initial clinical examination, the US examination was performed prospectively in each of the 109 patients by two otorhinolaryngologists (consultant and fellow) using a 10-MHz head (type EUP-L34T; Hitachi Medical Corp., Tokyo, Japan; size: 3 × 1.4 cm) of a commonly used ultrasound device (type EUB-525RS; Hitachi Medical Corp., Tokyo, Japan). If the tumor lesion was too large for the ultrasound head or located deeper in the salivary gland, an additional US was performed with a 7.5-MHz probe (model no. 1409692 LH 302 Siemens Medizintechnik AG, D-90439 Nuernberg, Germany, size 8.5 × 1.2 cm) of a Sonoline SI 400 (Siemens Medizintechnik AG, D-90439 Nuernberg, Germany). Both otolaryngologists were blinded to the results of the CT-/MRI-scans or the patients' history. Final interpretations forecasting diagnosis from US were made by the two otolaryngologists in consensus.

### Surgical treatment

After the clinical diagnosis had been made, all 109 patients underwent surgery. In 18 cases the submandibular gland was removed. In two patients the tumor of the submandibular gland was suspected to be malignant, and a complete neck dissection was performed.

In 89 cases the patients underwent surgery of the parotid gland. In 59 patients a superficial (laterofacial) parotidectomy was sufficient to remove the tumor. Two of these procedures were revision surgeries. Twenty patients (cases) underwent total parotidectomy and 8 patients, a radical parotidectomy with removal of the facial nerve. Three further patients suffered from a so-called dumbbell tumor of the parotid gland that required combined parapharyngeal and parotid surgery.

### Data analysis

After US examination, both otorhinolaryngologists in each case had to give a statement about the malignant or benign nature of the lesion, and the correct diagnosis of the tumor entity, e.g., carcinoma, cystadenolymphoma in consensus. The statements of the observers were compared with the histological diagnosis.

CT/MRI reports also were compared with the histological diagnosis in terms of the nature of the lesion (malignant/benign) and the correct diagnosis.

Cases were rated correct when the result of the CT/MRI or US matched the histological diagnosis. If they failed to match, they were rated incorrect. Particularly the assessment of the correct diagnosis in malignant tumors proved to be difficult. If the two readers were unable to make a statement about the correct diagnosis, the cases were rated incorrect.

### Statistical analysis

The statistics program used was Statistical Product and Service Solution 12.0 for Windows (SPSS Inc., Chicago, Illinois, USA). The Chi-square test was used to compare results between the three diagnostic tools (CT, MRI and US). The level of significance was set at p < 0.05. Furthermore, specificity (defined as the proportion of true negatives correctly identified by the test indicating how often a tumor-like lesion is diagnosed correctly), sensitivity (defined as the proportion of true positives correctly identified by the test indicating how often a non-tumor-like lesion is diagnosed correctly) and the accuracy (defined as the proportion of true negatives and true positives correctly identified by the test; indicating the agreement between the preoperative diagnosis and the histological outcome) were calculated.

## Results

### CT/MRI prevalence

The total number of scans – MRI or CT – performed in 582 patients during the years 2000–2004 was n = 179. In order to compare the quality of the different scans the investigations had to fulfil the guidelines and technical standards of the ACR

Interestingly, about 64,3% (n = 114) of these scans met the inclusion criteria of the study according to the ACR whereas 35,7% (n = 64) of CT-scans fulfiled not the required standards (Table 1). The annual frequency of imaging procedures (CT or MRI) rose from 12.5% in 2000 to 26.3% in 2004 (Table 1).

**Table 1:** Total Percentage of CT/MRI scans from 2000 to 2004

Year	Patients	CT	MRI	CT and MRI	Percentage of CT/MRI scans
2000	123	20	11	31	25.1%
2001	151	19	11	30	19.8%
2002	101	13	15	28	27.6%
2003	112	28	18	46	41.0%
2004	95	26	18	44	46.2%
**Total**	**582**	**106**	**73**	**179**	**30.7%**

Year	Patients	CT*	MRI*	CT* and MRI*	Percentage of CT/MRI scans

2000	123	9	11	20	16.2%
2001	151	8	11	19	12.5%
2002	101	6	15	21	20.7%
2003	112	11	18	29	25.9%
2004	95	7	18	25	26.3%
**Total**	**582**	**41**	**73**	**114**	**19.8%**

*fulfiling the standard ACR = American college of Roentgenology

37% of the scans fulfiling the ACR standards (37%) were arranged by general practitioners and 63% by ENT-specialists (Table 2). In contrast, about 60% of CT scans not fulfiling the standards were arranged by general practitioners.

**Table 2:** Percentage of CT/MRI scans arranged by General practitioners and ENT-specialists

	Imaging not according to standard ACR arranged by	
Year	**GP**	**ENT-Sp**

2000	5 (12%)	6 (22%)
2001	7 (17%)	4 (15%)
2002	4 (15%)	2 (11%)
2003	10 (27%)	7 (22%)
2004	12 (29%)	7 (30%)
**Total**	**41 (61%)**	**26 (39%)**

	Imaging according to standard of ACR arranged by	

Year	**GP**	**ENT-Sp**

2000	7 (35%)	13 (65%)
2001	9 (47%)	10 (53%)
2002	9 (43%)	12 (57%)
2003	12 (41%)	17 (59%)
2004	5 (25%)	20 (75%)
**Total**	**42 (37%)**	**72 (63%)**

At least 109 of 582 patients with a palpable suspected lesion of the salivary gland who had undergone CT or MRI, meeting the inclusion criteria for CT-or MRI-scan, prior to their first visit to our outpatient department underwent surgery and were enrolled for the further study.

### Annual distribution of benign and malignant tumors and the operations

In order to detect differences in the distribution of benign and malignant tumors over the years, we analyzed the average grade of malignancy, based on the results of postsurgical histopathology of 109 patients. No major differences were noted between the years of investigation in respect of the nature of tumors and the operations performed (Table 3). The maximum percentage of malignant tumors was observed in the year 2000 (30%) while the minimum percentage was seen in the year 2002 (Table 3). The majority of the operations were performed in the year 2001 (37%) (Table 3).

**Table 3:** Percentage of benign and malignant tumors and performed operations in 109 patients from 2000 to 2004

	**Tumors**		**Operations**		
Year	Benign	Malignant	Extirpation of the submandibular gland	Superficial parotidectomy	Total/radical parotidectomy; Neck dissection

2000	14 (70%)	6 (30%)	4 (20%)	10 (50%)	6 (30%)
2001	14 (74%)	5 (26%)	4 (21%)	8 (42%)	7 (37%)
2002	15 (75%)	5 (25%)	4 (20%)	11 (55%)	5 (25%)
2003	19 (73%)	7 (27%)	3 (12%)	15 (58%)	8 (30%)
2004	17 (71%)	7 (29%)	3 (13%)	15 (66%)	6 (26%)
**Total**	**79 (72%)**	**30 (28%)**	**18 (17%)**	**59 (54%)**	**32 (29%)**

### Histological results

The histological findings revealed that a benign tumor had been removed in 79 patients (72.5%) and a malignant tumor in 30 patients (27.5%). Pleomorphic adenoma was the most common benign tumor in 30 cases (27.5%), followed by cystadenolymphoma in 18 patients (6.1%). Among malignant lesions, adenocarcinoma was the most common (9 cases; 8.3%), followed by lymphoma (5 cases; 4.6%).

### Assessment of the tumor entity

Descriptive statistical analysis (specificity, sensitivity, accuracy) of the lesions revealed that CT and MRI delivered similar results as did the ultrasound examination (Table 4). US achieved in our study a sensitivity of 88%, a specificity of 54% and an accuracy of 79%. MRI investigations showed a sensitivity of 98%, a specificity of 52% and an accuracy of 84% The Chi-square test showed no statistically significant difference between CT/MRI and the ultrasound examination (Table 4). As expected, three dumbbell tumors were only seen on MRI or CT and could not be detected on US, even not with the use of a 7.5-MHz probe.

**Table 4:** Assessment of the benign or malignant nature of the lesion

**Radiological assessment**	**Histology**			**Sensitivity**	**Specificity**	**Accuracy**
	benign	malignant	total			

Ultrasound*						
Benign	71	13	84	88%	54%	79%
Malignant	10	15	25			
Total	81	28	109			
CT*						
Benign	31	3	34	91%	57%	85%
Malignant	3	4	7			
Total	34	7	41			
**Ultrasound and CT**						
Benign	30	3	33	88%	57%	83%
Malignant	4	4	8			
Total	34	7	41			
MRI*						
Benign	49	11	60	98%	52%	84%
Malignant	1	12	13			
Total	50	23	73			
**Ultrasound and MRI**						
Benign	43	11	54	86%	52%	75%
Malignant	7	12	19			
Total	50	23	73			

* Chi-square analysis: MRI versus Ultrasound χ^2 ^= 0.335; p = ns; CT versus Ultrasound χ^2 ^= 0.831; p = ns

### Assessment of the correct diagnosis

For the diagnosis of tumor-like lesions, CT and US yielded nearly the same results in respect of the correct diagnosis. In comparison, MRI proved superior to US (Table 5). The Chi-square test showed no significant difference between CT and US or MRI and US.

**Table 5:** Assessment of the correct diagnosis: All tumors (n = 109)

**Radiological assessment**	**Histological diagnosis**		
	**correct**	**incorrect**	**total**

Ultrasound*	49	60	109
CT*	16	25	41
Ultrasound and CT	19	22	41
MRI*	34	39	73
Ultrasound and MRI	31	42	73

* Chi-square analysis: MRI versus Ultrasound χ^2 ^= 0.719; p = ns; CT versus Ultrasound χ^2 ^= 0.449; p = ns

Dividing the analysis of correct diagnoses into benign and malignant tumors, it was found that in benign tumors the correct diagnosis was drawn in many cases by US/MRI and CT. The ultrasound examination seemed to be slightly superior to MRI and CT, although the statistical analysis revealed no significant differences (Table 6). In contrast, in malignant tumors it was possible to forecast the correct diagnosis only in a few cases. Here, MRI seemed to be slightly superior to the ultrasound examination while the poorest results were seen on CT (Table 7). The analysis of malignant tumors also revealed no significant difference between US, CT and MRI (Table 7).

**Table 6:** Assessment of the correct diagnosis: Benign tumors (n = 79)

**Radiological assessment**	**Histological diagnosis**		
	**correct**	**incorrect**	**total**

Ultrasound	45	34	79
CT	15	18	33
Ultrasound and CT	17	16	33
MRI	27	23	50
Ultrasound and MRI	25	25	50

* Chi-square analysis: MRI versus Ultrasound χ^2 ^= 0.572; p = ns; CT versus Ultrasound χ^2 ^= 0.651; p = ns

**Table 7:** Assessment of the correct diagnosis: Malignant tumors (n = 30)

**Radiological assessment**	**Histological diagnosis**		
	**correct**	**incorrect**	**total**

Ultrasound	4	26	30
CT	1	7	8
Ultrasound and CT	2	6	8
MRI	7	16	23
Ultrasound and MRI	6	17	23

* Chi-square analysis: MRI versus Ultrasound χ^2 ^= 0.702; p = ns; CT versus Ultrasound χ^2 ^= 0.562; p = ns

## Discussion

In recent years, an increase in the number of CT and MRI scans has been recorded for first-line diagnosis in patients with tumor-like lesions of the salivary glands at the outpatient care of an ENT-department (university hospital). 30.7% of the patients with tumor-like lesions in salivary glands underwent an imaging procedure – MRI or CT – prior to US and in about 11.0% of the patients CT scans lack standards like intravenous contrast or a slice thickness of 5 mm or less. Besides data presentation, the present study has been performed to highlight several issues explaining this phenomenon. Especially, one issue addresses the key question, whether MRI, CT and US devices of the newer generation were more valid to deduct the correct diagnosis with special focus on the nature of the tumor lesion (benign/malignant).

According to our study population, the percentage of CT and MRI scans with standard quality for first-line diagnosis in patients with tumor-like lesions of the salivary glands ranged from about 12% in the years 2000/1 to 26% in the years 2003/4. However, neither spectrum of operations nor the percentage of malignant tumors in our study population differed significantly. Within this context, the behaviors of general practitioners and ENT-specialists to recommend imaging procedures during the first visit of patients in their offices has been investigated. Nearly 37% of those scans, who met the criteria of ARS and nearly 61% of those CT scans that met not the criteria of ARS were arranged by general practitioners. In contrast, 63% of imaging, who met the criteria of ARS were arranged by ENT-specialists. We hypothized that both groups of medical doctors lack information and experience to choose a correct diagnostic tool. Obviously they took the conclusion that CT and MRI scans were the superior tool to detect tumor like lesion in salivary glands.

Thus, in order to examine the diagnostic value of CT and MRI versus US, our study patients with already performed imaging underwent an US-analysis. Results of imaging devices were compared to postoperative histology of the pathology. To minimize the influence of technical factors on the outcome of CT and MRI examinations, the inclusion criteria for imaging, as stated in the material and methods section, had to be fulfiled.

Benign tumors were found in 72% of patients while malignant tumors were demonstrated in 28%, both in accordance with the literature. Pleomorphic adenoma was the most common entity among benign tumors while adenocarcinoma was most common among malignant lesions [4].

In assessing a tumor entity-maligne or benigne, US achieved in our study a sensitivity of 88%, a specificity of 54% and an accuracy of 79%. These results are comparable to those in the literature, which report an accuracy of 82.3% for US [8]. MRI investigations showed a sensitivity of 98%, a specificity of 52% and an accuracy of 84%. According to Takashima et al., MRI achieved a sensitivity of 60%, a specificity of 88% and an accuracy of 81% in the assessment of tumor malignancy [18]. Although MRI was slightly superior to US in the present study, no statistical significant difference was detected between US versus MRI or CT.

Furthermore, our results ruled out that superficial tumors of the parotid gland are well assessed by US. MRI provides here no additional information about the malignancy, size, and margins of the tumor as discussed in literature [17]. Very large tumors or those in a far medial or parapharyngeal location tend to cause difficulties [10,16]. In the present study, none of the dumbbell tumors could be visualized on the ultrasound examination despite the use of a 7.5-MHz ultrasound probe, which is able to better visualize deeper portions of the parotid gland than the 10-MHz probe. However, all dumbbell tumors could be assessed well on CT, and particularly on MRI [9] .

In the present study, CT and MRI were comparable in respect of demonstrating benign and malignant entities (CT: sensitivity 91%, specificity 57%, accuracy 78%; MRI: sensitivity 98%, specificity 52%, accuracy 84%). Koyuncu et al. described the similar results, indicating no significant differences between MRI and CT according to tumor location, invasion, and margin characteristics [12]; both imaging techniques provided the same information for pre-surgical planning. However, in contrast, other studies came to the conclusion that MRI is superior to CT [3] or that MRI and ultrasound, both achieve a more accurate diagnosis [7][13][15] .

Forecasting the correct diagnosis of tumor-like lesions of the salivary gland proved to be difficult. Divided into benign and malignant lesions, the forecast of the correct diagnosis was particularly weak for malignant tumors. In benign tumors, US could forecast the correct diagnosis in 45 of 79 cases, MRI in 27 of 50 cases, and CT in 15 of 33 cases. In malignant lesions US could forecast the correct diagnosis in 4 of 30 cases, MRI in 7 of 23 cases, and CT in 1 of 8 patients. These observations concur with published data, where in benign tumors the correct diagnosis could be established by US in 54%, whereas in malignant tumors, forecasting the diagnosis of salivary gland tumors is difficult with any imaging technique [8,11].

In summary, the increase in the number of CT and MRI scans performed in recent years to diagnose a tumor like lesion in salivary glands can not be explained by arguing that CT or MRI represents a superior diagnostic tool. None of the examined imaging procedures MRI, CT or US is superior to diagnose a tumor in the salivary glands, but all imaging procedures allow detecting a tumor. None of the procedures allows a safety forecast for the correct diagnosis of a maligne tumor entity.

## Conclusion

The authors recommend that the ENT specialists should decide whether additional imaging gives further information besides history and clinical examination. When imaging is required, US should be firstly taken into consideration, as it provides different advantages: no radiation, low costs, use of fine needle biopsy and mostly the same information as other imaging procedures.

Only in special cases, such as a tumor in a deep location, a dumbbell tumor or bone infiltration, a MRI or CT investigation should be performed. Scans had to fulfil the most common standards. Updating or revising the current preoperative diagnostic management of tumor-like lesions of the salivary glands is deemed necessary.
